# Disparities and barriers of health facility delivery following optimal and suboptimal pregnancy care in Nigeria: evidence of home births from cross-sectional surveys

**DOI:** 10.1186/s12905-023-02364-6

**Published:** 2023-04-25

**Authors:** Oyewole K. Oyedele

**Affiliations:** 1 International Research Center of Excellence, Institute of Human Virology, Nigeria (IHVN), Abuja (FCT), Nigeria; 2 Department of Epidemiology and Medical Statistics, Faculty of Public Health, College of Medicine, University of Ibadan, Ibadan, Nigeria

**Keywords:** Antenatal care, Home delivery, Healthcare facility, Women, Multivariable regression

## Abstract

**Background:**

Despite uptake of antenatal care (ANC), 70% of global burden of maternal and child mortality is prevalent in sub-Saharan Africa, particularly Nigeria, due to persistent home delivery. Thus, this study investigated the disparity and barriers to health facility delivery and the predictors of home delivery following optimal and suboptimal uptake of ANC in Nigeria.

**Methodology:**

A secondary analysis of 34882 data from 3 waves of cross-sectional surveys (2008–2018 NDHS). Home delivery is the outcome while explanatory variables were classified as socio-demographics, obstetrics, and autonomous factors. Descriptive statistics (bar chart) reported frequencies and percentages of categorical data, median (interquartile range) summarized the non-normal count data. Bivariate chi-square test assessed relationship at 10% cutoff point (*p* < 0.10) and median test examined differences in medians of the non-normal data in two groups. Multivariable logistic regression (Coeff plot) evaluated the likelihood and significance of the predictors at *p* < 0.05.

**Results:**

46.2% of women had home delivery after ANC. Only 5.8% of women with suboptimal ANC compared to the 48.0% with optimal ANC had facility delivery and the disparity was significant (*p* < 0.001). Older maternal age, SBA use, joint health decision making and ANC in a health facility are associated with facility delivery. About 75% of health facility barriers are due to high cost, long distance, poor service, and misconceptions. Women with any form of obstacle utilizing health facility are less likely to receive ANC in a health facility. Problem getting permission to seek for medical help (aOR = 1.84, 95%CI = 1.20–2.59) and religion (aOR = 1.43, 95%CI = 1.05–1.93) positively influence home delivery after suboptimal ANC while undesired pregnancy (aOR = 1.27, 95%CI = 1.01–1.60) positively influence home delivery after optimal ANC. Delayed initiation of ANC (aOR = 1.19, 95%CI = 1.02–1.39) is associated with home delivery after any ANC.

**Conclusions:**

About half of women had home delivery after ANC. Hence disparity exist between suboptimal and optimal ANC attendees in institutional delivery. Religion, unwanted pregnancy, and women autonomy problem raise the likelihood of home delivery. Four-fifth of health facility barriers can be eradicated by optimizing maternity package with health education and improved quality service that expand focus ANC to capture women with limited access to health facility.

## Introduction

Optimal utilization of Reproductive Health (RH) services that includes maternal and perinatal healthcare addresses the physical, mental and social wellbeing of the birthing population before, during and after childbirth [1, 2]. Thus, access to quality RH service is a major determinant of positive birth outcomes and to ensure mother and child survival [3, 4]. However, low uptake of maternal and newborn care services provided by skilled professionals in healthcare facility particularly in the low-or middle-income countries (LMICs) tends to increase the risk of maternal and child mortality which has been a major public health concern [5–8].

The burden of maternal and child mortality is highest in developing countries with sub-Saharan Africa (SSA) accounting for about 70% and Nigeria is among the top five countries mostly affected in the world [9, 10]. Evidently, due to the low use of the reproductive health services. For instance, only 39% (3% increase from 2013) of the women utilized health facility at delivery and 43% (4% increase from 2008) utilized the service of skilled health professional during childbirth [11, 12]. According to the World Health Organization, maternal mortality ratio is as high as 917 deaths per 100,000 livebirths in Nigeria and contrary to the underreported estimates (512 deaths/100,000 livebirths) from the recent Nigerian Demographic and Health Survey (NDHS) [12, 13]. The recent NDHS further reported that 67% of women attended at least one ANC service while only 57.4% received optimal care (i.e. four or more ANC visits) and thus implies that about 10% of the visit were suboptimal (less than the require four visits) while 33% of pregnant women do not receive any ANC service [12, 14]. Also, Pregnancy-related mortality ratio (PRMR) only reduce from 576 deaths/100,000 livebirths in 2006–2013 to 556 deaths/100,000 livebirths in 2011–2018 while about one in every 25 livebirth dies before first month birthday [12].

These statistics pointed to low utilization of maternal health service particularly at delivery, which undermine the achievement of sustainable development goal 3 (SDG-3) targeted towards reducing maternal and newborn mortality to 70 maternal death/100,000 livebirths and 12 newborn deaths/1000 livebirths by 2030 respectively [15]. Though these mortality estimates have reduced overtime, but it took too long following the millennium development goal to achieve such small decline as the global health communities count down towards the 2030 targets and thus, question the realization of the 2030 SDG-3 in Nigeria.

Literatures has widely documented that sociodemographic, reproductive, and other maternal health factors are associated with utilization of maternal health care services [16–21] in SSA. This includes the antenatal care uptake [14, 22, 23], skilled provider use [24–26], and utilization of postnatal care services [27–29]. Also, women continuity of the maternity health care continuum (from antepartum to postpartum) has been studied [30–32]. Studies disclosed that women age [33–35], maternal education [36–38], residence[35–37], wealth [35–39], parity [36, 37, 39, 40], ANC initiation and contacts [34, 36–41], are associated with maternal utilization of healthcare facility at delivery. However, studies rarely assess the barriers of facility delivery [42], and the resultant predictors of non-facility delivery in Nigeria [43, 44]. To the best of the author’s knowledge, there is no known national study on predictors of home delivery following pregnancy care in Nigeria.

Meanwhile women utilization of healthcare facility during pregnancy and childbirth remains an ideal prevention mechanism to reduce the risk of adverse birth outcome and increase the chance of positive pregnancy outcome after antenatal care [4, 45]. However, the prevalence of institutional delivery is below the level of expectation as per WHO recommendation and even below the ANC prevalence in Nigeria [12, 30]. This indicate dropout in the women continuation to the use of health facility at childbirth after ANC as it remains to be seen, how the level of effort geared towards increasing uptake of antenatal care service translate into improved utilization of healthcare facility at delivery and subsequently help to curb the risen burden of maternal and child health.

Thus, stressing the need to study and address the gap (barriers) in the utilization of healthcare facility at delivery following antenatal care uptake. Hence, this study investigates the disparities and blockages of institutional births after suboptimal and optimal use of antenatal care service. The following research questions were answered: Any disparity in the prevalence of home and facility delivery among ANC attendees? What are the predictors of non-institutional delivery after optimal and suboptimal ANC visits? What are the barriers to health facility delivery after ANC visits? The study findings will inform policy strategy to improve women turnup and uptake of reproductive health services toward improving maternal and child health indicators for attainment of SDGs.

## Materials and methods

### Study design, data source and area

The study is a secondary analysis of data from three successive cross-sectional surveys conducted by the Nigerian Demographic Health Survey (NDHS) in 2008, 2013 and 2018. NDHS is a population-based nationally representative survey that is routinely collected across the enumeration areas in the states based on sampling strategy. Following the inception in 1990, the survey has been collected every 5 years (2003, 2008, 2013 and recently 2018) in Nigeria. Nigeria comprises of 6 geopolitical zones (Northcentral, Northeast, Northwest, Southeast, South-south and Southwest) which are divided into 36 states and the federal capital territory and subdivided into 774 local government areas which serve as the closest administrative unit to the communities [12].

### Sampling technique and participants

**Fig. 1:**
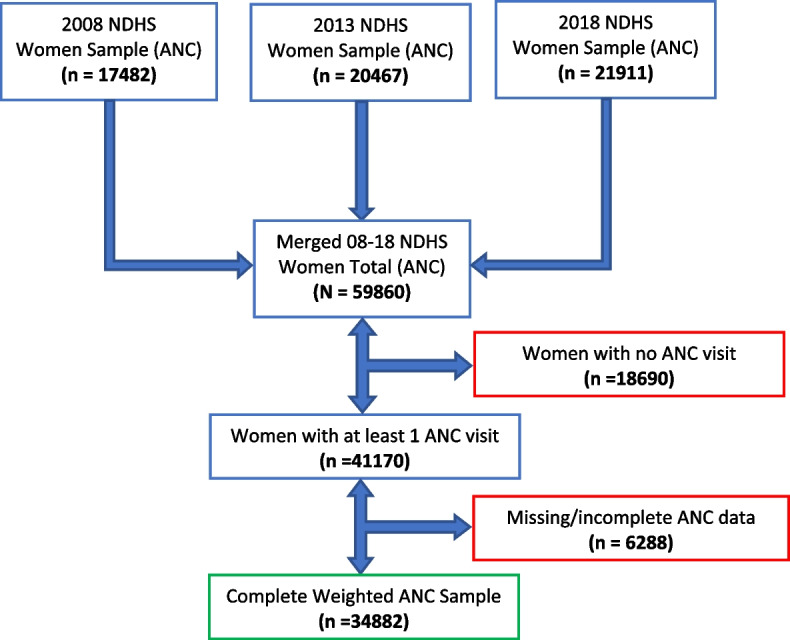
Data flow diagram for the selection of final analysis sample (NDHS 2008–2018)

A similar multistage sampling technique was adopted in the 3 waves of the survey between 2008 and 2018. Such that the selected local government areas from the 36 states and the federal capital territory make up the first stage sampling and the subsequent selection of rural and urban enumeration areas (primary sampling unit) from the administrative unit was the second stage sampling. Household sampling frame of the National Population and Housing Census was adopted to select households (third stage) as the primary sampling unit (cluster). 33385, 38984 and 41821 women participants were reportedly interviewed in the 2008, 2013 and 2018 surveys respectively [11, 12, 46]. Complete responses from the women of reproductive age (15–49 years) who had at least a birth in the five years preceding the survey and who had attended at least one antenatal care visit (34882) were included in the data analysis and otherwise excluded (Fig. 1).

### Measures of outcome

The outcome variable is the place of delivery at last childbirth which was measured from the question on type of place of delivery of index child [11, 12, 46]. Multiple responses (respondent home, government hospital, government health center, private hospital/clinic, and other homes) received fall under two categories and were subsequently classified as illustrated below.

$$\mathbf P\mathbf l\mathbf a\mathbf c\mathbf e\boldsymbol\;\mathbf o\mathbf f\boldsymbol\;\mathbf d\mathbf e\mathbf l\mathbf i\mathbf v\mathbf e\mathbf r\mathbf y=\left\{\begin{array}{l}1,\;Health\;facility\;i.e.\;delivery\;in\;an\;healthcare\;institution\\0,\;Elsewhere\;i.e.\;delivery\;at\;home\;(non-institutional)\end{array}\right.$$

The outcome data was further grouped by the optimality of antenatal care. ANC was optimal when four or more ANC services were received during pregnancy and suboptimal when less than 4 ANC service were received. This was based on the WHO recommendation of 4 + ANC and not the recently recommended 8 + ANC since the 2008–2018 NDHS framework was operationalized by the 4 + ANC and all the pregnancies care services investigated in this study had occur prior to devising the strategy to adopt the 8 + ANC through orientation packages in Nigeria [30, 47, 48].

### Explanatory factors

Selection and inclusion of independent variables in this study were based on similar factors considered in previous literatures on maternal healthcare service utilization [33–41]. This was defined under the broad categories as; Demographic characteristics, pregnancy and childbirth factors, socio-economic features that includes wealth quintiles, and autonomous factors [25, 30].

#### Demographic characteristics

Age-group (15–24, 25–34, 35–49 years), education (no formal education, primary, secondary, tertiary), Marital status (married, unmarried), partner education (no formal education, primary, secondary, tertiary), place of residence (urban rural), region (northcentral, northeast, northwest, southeast, south-south, southwest), religion (Christianity, Islam, traditional/other).

#### Socio-economic characteristics

Occupation (unemployed, employed), wealth (poorest, poorer, average, richer, richest), media exposure (no, yes), covered by health insurance (no, yes), medical help-money (not a big problem, big problem).

#### Pregnancy and childbirth factors

Wanted pregnancy (then, later, no more), birth order (1, 2, 3, 4, 5 +), prenatal provider (unskilled, skilled), SBA use (unskilled, skilled), time of ANC initiation (1^st^, 2^nd^, 3^rd^), ANC place (home, health facility).

#### Healthcare accessibility and women autonomous factors

Medical help-permission (not a big problem, big problem), medical help-distance (not a big problem, big problem), health decision maker (partner alone, joint decision, woman alone).

### Data management and statistical analysis

Data management began by variable validation and comparison for each of the extracted 2008, 2013 and 2018 survey data from the open repository of (dhsprogram.com). Data merging and cleaning removed incomplete/missing data (Fig. 1). The weighted indices included in the Women recode sample of the NDHS was applied to weight the merged data. The svyset command adjusted for the disproportionate population size and account for weighting, clustering and stratification design of the surveys using Stata version 17.0 (Stata Corp, Texas, USA). Variable (Union type) leading to multicollinearity was removed and substituted with marital status which have a weaker correlation.

Descriptive analysis of the complete (non-missing) data was initially performed and the corresponding frequency and percentage were reported for each of the categorical variable per ANC group (optimal and suboptimal). Women proportion based on the barriers of facility delivery were reported and the median (IQR) statistics of the non-normally distributed count data (number of ANC visits) disaggregated by each barrier group were reported. Type of place of health facility delivery was coded 1 if delivery was in a health facility and 0 if elsewhere. Clustered bar chart describes women proportion of facility and non-facility delivery use by ANC status.

Bivariate chi-square analysis was subsequently performed by setting the cutoff point at 10% (i.e., *p* < 0.10) to determine the set of important explanatory factors that will be included in the multivariate analysis. The Pearson chi-square test was conducted for the suboptimal and optimal as well as the combined group. Pearson chi-square statistics (*p* < 0.001 throughout) was reported as none of the 20% of the expected cell count was less than 5. Also, Median test was performed to assess difference in medians (number of antenatal care visits) between two groups of health facility delivery barriers since the count data is not a normal but rather a rightly skewed distribution. Hence all the factors reported in the bivariate analysis were included in the multivariate analysis.

Multivariable binary logistic regression model was then fitted to evaluate the likelihood and significance of the important independent variables in the bivariate analysis above. This was performed for the suboptimal (model 1) and optimal (model 2) and the combined group (model 3). Adjusted and crude odds ratio were reported when other factors were included and excluded in the models respectively. The adjusted logit regression was equally carried out to assess the probability of health facility ANC among women with facility delivery barriers and the Coeff plot showing the corresponding adjusted odds ratio was reported. All statistical analysis were performed at 5% level of error tolerance (95% confidence interval) in Stata 17.0 (Stata Corp, Texas, USA).

### The multivariable model

Multiple binary logistic regression was fitted to assess the probability and significance of the explanatory factors predicting the outcome (non-facility delivery) which is based on the binary response $\left[P\left(Y_i=1\;if\;helath\;facility\;delivery\right),P\left(Y_i=0\;if\;home\;birth\right)\right]$ such that the binary regression estimate due to the shape parameter of the logistic curve is obtainable under the maximum likelihood estimator, and not the least square estimator (line of fit) in the linear regression [30, 49]. The multivariable binary logistic regression equations for the outcome ‘Y’ as a linear combination of the regression coefficients ‘β’ and the predictors ‘X’ are illustrated below.

1 $$Y =F \left(\beta X\right)$$

2 $${Y}_{i}= \mathrm{ln}\left(\frac{p}{1-p}\right)= {\beta }_{0}+ {\beta }_{1}{X}_{1i}+\dots + {\beta }_{p}{X}_{pi}+ \varepsilon$$

3 $$E\left({Y}_{i}\right)={p}_{i}=\frac{exp\left({\beta }_{0}+{\beta }_{1}{x}_{pi}+\dots +{\beta }_{p}{x}_{pi}\right)}{1+exp\left({\beta }_{o}+{\beta }_{1}{x}_{1i}+\dots +{\beta }_{p}{x}_{pi}\right)}$$

where: $\mathrm{ln}\left(\frac{\mathrm{p}}{1-\mathrm{p}}\right)$ is the log odds [p is the probability of success (i.e., utilizing healthcare facility at delivery) and 1-p is the failure probability (i.e., home/non-facility delivery)].${\beta }_{0}$ is the logistic regression constant or intercept.${\beta }_{1}+\dots + {\beta }_{p}$ are the px1 vector of the regression coefficient or slopes.

${X}_{i1}+\dots +{X}_{ip}$ are the nxp matrix of the explanatory variables predicting the log odds in the model.

## Results

### Type of place of delivery disaggregated by optimality of ANC Visits

**Fig. 2:**
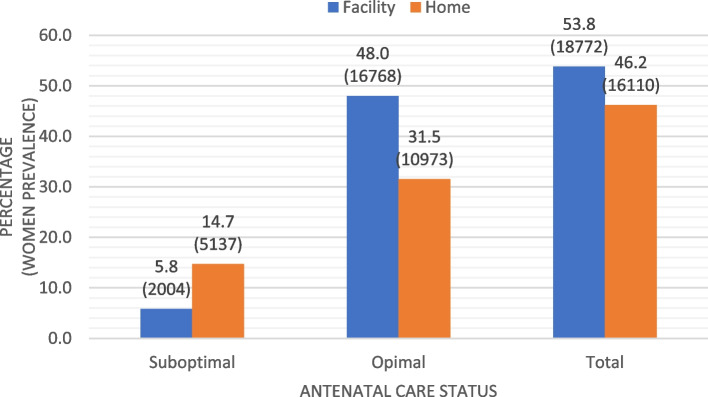
Prevalence of facility and non-facility delivery by antenatal care status

Figure 2 shows the prevalence of home and facility delivery by ANC status. Of the weighted women total (34882), 53.8% (18772) had facility delivery while 46.2% of the women had non-facility delivery (14.7% in suboptimal ANC group and 31.5% in Optimal ANC group). 5.8% in the suboptimal ANC group versus 48.0% in the optimal ANC group had facility delivery respectively (Fig. 2).

### Descriptive statistics of women characteristics by ANC status

Table 1 shows the descriptive statistics of women respondent based on ANC optimality. Of the total weighted sample (34882), 50.2% (17503) of the women are of reproductive age (25-34 years) with 9.5% and 40.7% having suboptimal and optimal ANC visits. Most (36.9%) women have secondary education while least proportion (10.5%, with 9.9% in optimal group) have tertiary education. About 97% (33792) are married and majority (39.3%) of their partners have secondary education (33.6% and 5.7% in optimal and suboptimal ANC class respectively). 53.6%(18694) of the women resides in the rural while 46.4% (16188) are urban dwellers (Table 1). Most women (25.1%) are in the highest wealth class while few (12.6%) are in the lowest wealth class. 55.6% (47.9% in optimal ANC group) are exposed to mass media, 88.6% (30913) desired the pregnancy, 7.8% wanted it later and 3.6% wanted it no more (Table 1). 34.3% (11956) have had at least 4 births while 17.3% (6025) are primiparous. Only 2.5% (2.3% in optimal group) are covered by health insurance with money for medical help being a big problem for 43.2% (15075). About 52% (18065) reported that health decisions were made by their partner alone while 8.4% have problem getting permission for medical help (Table 1). About 91% and 58.4% of respondent had skilled attendants at ANC and delivery respectively. Most ANC was initiated in the 2nd trimester (62.6%) and in a health facility (95.3%).

**Table 1:** Descriptive statistic by status of ANC

Characteristics	Suboptimal ANC n(%)	Optimal ANC n(%)	All ANC n(%)
**Age group**			
15–24	2031 (5.8)	5817 (16.7)	7848 (22.5)
25–34	3309 (9.5)	14194 (40.7)	17503 (50.2)
35–49	1801 (5.2)	7730 (22.2)	9531 (27.3)
**Education**			
No formal education	3812 (10.9)	7086 (20.3)	10898 (31.2)
Primary	1549 (4.4)	5922 (17.0)	7471 (21.4)
Secondary	1568 (4.5)	11288 (32.4)	12856 (36.9)
Tertiary	212 (0.6)	3445 (9.9)	3657 (10.5)
**Marital Status**			
Married	6979 (20.0)	26813 (76.9)	33792 (96.9)
Unmarried	162 (0.5)	928 (2.6)	1090 (3.1)
**Partner education**			
No formal education	2921 (8.4)	4992 (14.3)	7913 (22.7)
Primary	1455 (4.2)	5333 (15.3)	6788 (19.5)
Secondary	2007 (5.7)	11713 (33.6)	13720 (39.3)
Tertiary	758 (2.2)	5703 (16.3)	6461 (18.5)
**Place of residence**			
Urban	2102 (6.0)	14086 (40.4)	16188 (46.4)
Rural	5039 (14.5)	13655 (39.1)	18694 (53.6)
**Region**			
Northcentral	1314 (3.8)	4055 (11.6)	5369 (15.4)
Northeast	1876 (5.4)	3495 (10.0)	5371 (15.4)
Northwest	2703 (7.8)	5972 (17.1)	8675 (24.9)
Southeast	452 (1.3)	36,664 (10.5)	4116 (11.8)
South-south	463 (1.3)	3314 (9.5)	3777 (10.8)
Southwest	333 (1.0)	7241 (20.7)	7574 (21.7)
**Religion**			
Christianity	2237 (6.4)	14580 (41.8)	16817 (48.2)
Islam	4836 (13.9)	12970 (37.2)	17806 (51.1)
Traditional/other	68 (0.2)	191 (0.5)	259 (0.7)
**Occupation**			
Unemployed	2468 (7.1)	6807 (19.5)	9275 (26.6)
Employed	4673 (13.4)	20934 (60.0)	25607 (73.4)
**Wealth**			
Poorest	1761 (5.0)	2648 (7.6)	4409 (12.6)
Poorer	2002 (5.7)	4221 (12.1)	6223 (17.8)
Average	1649 (4.7)	5729 (16.4)	7378 (21.1)
Richer	1174 (3.4)	6955 (19.9)	8129 (23.3)
Richest	555 (1.6)	8188 (23.5)	8743 (25.1)
**Media exposure**			
No	4452 (12.8)	11007 (31.6)	15459 (44.4)
Yes	2689 (7.7)	16734 (47.9)	19423 (55.6)
**Wanted pregnancy**			
Then	6452 (18.5)	24461 (70.1)	30913 (88.6)
Later	501 (1.5)	2202 (6.3)	2703 (7.8)
No more	188 (0.5)	1078 (3.1)	1266 (3.6)
**Birth order**			
1	1066 (3.1)	4959 (14.2)	6025 (17.3)
2	1095 (3.1)	5322 (15.3)	6417 (18.4)
3	1070 (3.1)	4595 (13.1)	5665 (16.2)
4	913 (2.6)	3905 (11.2)	4819 (13.8)
5 +	2997 (8.6)	8960 (25.7)	11956 (34.3)
**Covered by health insurance**			
No	7066 (20.3)	26929 (77.2)	33995 (97.5)
Yes	75 (0.2)	812 (2.3)	887 (2.5)
**Medical help-permission**			
Not a big problem	6358 (18.2)	25602 (73.4)	31960 (91.6)
Big problem	783 (2.3)	2139 (6.1)	2922 (8.4)
**Medical help-money**			
Not a big problem	3420 (9.8)	16387 (47.0)	19807 (56.8)
Big problem	3721 (10.7)	11354 (32.5)	15075 (43.2)
**Medical help-distance**			
Not a big problem	4769 (13.7)	21546 (61.7)	26315 (75.4)
Big problem	2372 (6.8)	6195 (17.8)	8567 (24.6)
**Health decision maker**			
Partner alone	4816 (13.8)	13249 (38.0)	18065 (51.8)
Joint decision	1847 (5.3)	11509(33.0)	13356 (38.3)
Woman alone	478 (1.4)	2983 (8.5)	3461 (9.9)
**Prenatal provider**			
Unskilled	1134 (3.2)	2051 (5.9)	3186 (9.1)
Skilled	6007 (17.3)	25690 (73.6)	31696 (90.9)
**SBA use**			
Unskilled	495 6(14.2)	9539 (27.4)	14495 (41.6)
Skilled	2185 (6.3)	18202 (52.1)	20387(58.4)
**Time of ANC initiation**			
1^st^	642 (1.9)	8169 (23.4)	8811 (25.3)
2^nd^	3882 (11.1)	17968 (51.5)	21850 (62.6)
3^rd^	2617 (7.5)	1604 (4.6)	4221 (12.1)
**ANC place**			
Home	306 (0.9)	1326 (3.8)	1632 (4.7)
Health Facility	6835 (19.6)	26415 (75.7)	33250 (95.3)
Total	7141 (20.5)	27741 (79.5)	34882 (100)

*ANC* Antenatal Care, *SBA* Skilled Birth Attendant

### Bivariate analysis of the relationship between home delivery and women characteristics

The bivariate association between home delivery and respondent characteristics are shown in Table 2. About 21.9% of women (25–34 years) had home birth compared to 28.3% that had facility birth. 11.2% and 10.8% of women with secondary education who had suboptimal and optimal ANC delivered at home respectively. 54.5% (3889) of women in rural who had suboptimal ANC had home birth compared to the 16.1% that had facility delivery (Table 2). 54.2% of Muslim women that had suboptimal ANC delivered at home while 25.6% that received optimal ANC had home birth. 66.4% (4740) of women in the suboptimal group who desired pregnancy had home birth while 24.0% had facility birth (Table 2). 51.5% of women in suboptimal ANC group whose partner made their health decision had home birth compared to 16% that had facility birth. 52.1% of women had at least one ANC provided by skilled attendants in a health facility. About 40% of women who initiated suboptimal and optimal ANC in 2^nd^ trimester had home and facility delivery respectively (Table 2). Overall, 79.1% and 28.1% of the women with suboptimal ANC had home and facility delivery respectively, while 39.6% and 60.4% of the women with optimal ANC had home and facility delivery respectively. All the women characteristics were significant at *p* < 0.001 based on Pearson chi-square test (Table 2).

**Table 2:** Bivariate chi-square analysis of home delivery and maternal factors by ANC status

Characteristics	Suboptimal ANC		Optimal ANC		All ANC		*p*-value
	Home n(%)	Facility n(%)	Home n(%)	Facility n(%)	Home n(%)	Facility n(%)	
**Age group**							< 0.001^a^
15–24	1446(20.3)	584(8.2)	2814(10.1)	3002(10.8)	4261(12.2)	3587(10.3)	
25–34	2351(32.9)	957(13.4)	5285(19.1)	8909(32.1)	7637(21.9)	9866(28.3)	
35–49	1338(18.7)	463(6.5)	2874(10.4)	4857(17.5)	4212(12.1)	5319(15.2)	
**Education**							< 0.001^a^
No formal education	3203(44.8)	609(8.5)	5012(18.1)	2074(7.5)	8214(23.5)	2684(7.7)	
Primary	1078(15.1)	471(6.6)	2674(9.6)	3248(11.7)	3753(10.8)	3719(10.7)	
Secondary	797(11.2)	770(10.8)	2991(10.8)	8297(29.9)	3789(10.9)	9068(26.0)	
Tertiary	58(0.8)	154(2.2)	296(1.1)	3149(11.3)	354(1.0)	3302(9.5)	
**Marital Status**							< 0.001^a^
Married	5037(70.5)	1942(27.2)	10651(38.4)	16162(58.2)	15688(44.9)	18103(51.9)	
Unmarried	99(1.4)	62(0.9)	332(1.2)	606(2.2)	422(1.2)	669(1.9)	
**Partner education**							< 0.001^a^
No formal education	2484(34.8)	436(6.1)	3541(12.8)	1450(5.2)	6026(17.3)	1886(5.4)	
Primary	1075(15.1)	380(5.3)	2354(8.5)	2979(10.7)	3429(9.8)	3359(9.6)	
Secondary	1187(16.6)	820(11.5)	3786(13.6)	7927(28.6)	4974(14.3)	8747(25.1)	
Tertiary	390(16.6)	368(5.2)	1292(4.7)	4412(15.9)	1681(4.8)	4780(13.7)	
**Place of residence**							< 0.001^a^
Urban	1247(17.4)	854(12.0)	3730(13.5)	10355(37.3)	4978(14.3)	11209(32.1)	
Rural	3889(54.5)	1150(16.1)	7243(26.1)	6413(23.1)	11132(31.9)	7563(21.9)	
**Region**							< 0.001^a^
Northcentral	753(10.5)	560(7.8)	1376(4.9)	2678(9.6)	2130(6.1)	3239(9.3)	
Northeast	1469(20.6)	407(5.7)	2166(7.8)	1329(4.8)	3635(10.4)	1737(4.9)	
Northwest	2272(31.8)	431(6.0)	4239(15.3)	1733(6.3)	6511(18.7)	2164(6.2)	
Southeast	184(2.6)	268(3.7)	555(2.0)	3109(11.2)	739(2.1)	3376(9.6)	
South-south	306(4.3)	156(2.2)	1167(4.2)	2147(7.7)	1473(4.2)	2304(6.6)	
Southwest	152(2.1)	181(2.5)	1470(5.3)	5772(20.8)	1622(4.2)	5953(17.1)	
**Religion**							< 0.001^a^
Christianity	1211(16.9)	1025(14.4)	3768(13.6)	10812(39.0)	4980(14.2)	11836(33.9)	
Islam	3868(54.2)	968(13.5)	7101(25.6)	5868(21.2)	10969(31.4)	6837(19.6)	
Traditional/other	57(0.8)	11(0.2)	104(0.4)	88(0.2)	161(0.5)	99(0.3)	
**Occupation**							< 0.001^a^
Unemployed	1857(26.0)	610(8.6)	3278(11.8)	3529(12.7)	5136(14.7)	4139(11.9)	
Employed	3729(45.9)	1394(19.5)	7695(27.8)	13239(47.7)	10974(31.5)	14633(41.9)	
**Wealth**							< 0.001^a^
Poorest	1528(21.4)	232(3.3)	2052(7.4)	596(2.1)	3580(10.3)	829(2.4)	
Poorer	1592(22.3)	410(5.7)	2684(9.7)	1537(5.5)	4276(12.3)	1947(5.6)	
Average	1140(15.9)	509(7.1)	2739(9.9)	2990(10.8)	3879(11.1)	3499(10.0)	
Richer	661(9.3)	513(7.2)	2198(7.9)	4758(17.2)	2859(8.2)	5271(15.1)	
Richest	215(3.0)	339(4.8)	1300(4.7)	6887(24.8)	1515(4.3)	7227(20.7)	
**Media exposure**							< 0.001^a^
No	1000(14.0)	3452(48.3)	5870(21.2)	5136(18.5)	9323(26.7)	6136(17.6)	
Yes	1004(14.1)	1684(23.6)	5103(18.4)	11632(41.9)	6787(19.5)	12636(36.2)	
**Wanted pregnancy**							< 0.001^a^
Then	4740(66.4)	1711(24.0)	9847(35.5)	14613(52.7)	14588(41.8)	16324(46.8)	
Later	286(4.0)	215(3.0)	792(2.9)	1411(5.1)	1078(3.1)	1626(4.7)	
No more	110(1.5)	78(1.1)	334(1.2)	744 (2.6)	444(1.3)	822(2.4)	
**Birth order**							< 0.001^a^
1	655(9.2)	410(5.7)	1513(5.5)	3446(12.4)	2168(6.2)	3856(11.1)	
2	756(10.6)	339(4.7)	1805(6.5)	3517(12.7)	2561(7.3)	3856(11.1)	
3	746(10.4)	324(4.5)	1611(5.8)	2984(10.7)	2357(6.8)	3308(9.5)	
4	636(8.9)	277(3.8)	1509(5.4)	2397(8.6)	2145(6.2)	2674(7.7)	
5 +	2343(32.8)	653(9.1)	4356(16.4)	4424(15.9)	6879(19.7)	5077(14.6)	
**Covered by health insurance**							< 0.001^a^
No	5104(71.4)	1961(27.5)	10859(39.2)	16069(57.9)	15964(45.8)	18031(51.7)	
Yes	32(0.5)	43(0.6)	114(0.4)	699(2.5)	146(0.4)	742(2.1)	
**Medical help-permission**							< 0.001^a^
Not a big problem	4524(63.4)	1834(25.7)	9925(35.8)	15678(56.5)	1449(41.4)	17512(50.2)	
Big problem	612(8.5)	170(2.4)	1048(3.8)	1090(3.9)	1661(4.8)	1260(3.6)	
**Medical help-money**							< 0.001^a^
Not a big problem	2385(33.4)	1035(14.5)	5940(21.4)	10447(37.6)	8325(23.9)	11483(32.9)	
Big problem	2751(38.5)	969(13.6)	5033(18.2)	6328(22.8)	7875(22.3)	7289(20.9)	
**Medical help-distance**							< 0.001^a^
Not a big problem	3283(46.0)	1486(20.8)	8062(29.1)	13483(48.6)	11346(32.5)	14969(42.9)	
Big problem	1853(25.9)	518(7.3)	2911(10.5)	3284(11.8)	4764(13.6)	3803(10.9)	
**Health decision making**							< 0.001^a^
Partner alone	3677(51.5)	1139(16.0)	6770(24.4)	6478(23.3)	10,448(30.0)	7617(21.8)	
Joint decision	1155(16.2)	692(9.7)	3275(11.8)	8234(29.7)	4430(12.7)	8926(25.6)	
Woman alone	304(4.3)	173(2.4)	928(3.4)	2056(7.4)	1232(3.5)	2229(6.4)	
**Prenatal provider**							< 0.001^a^
Unskilled	982(13.7)	152(2.1)	1587(5.7)	464(1.7)	2569(7.4)	616(1.7)	
Skilled	4154(58.2)	1852(26.0)	9386(33.9)	16304(58.7)	13541(38.8)	18156(52.1)	
**SBA use**							< 0.001^a^
Unskilled	4807(67.3)	148(2.1)	9080(32.7)	459(1.7)	13888(39.8)	608(1.7)	
Skilled	323(4.6)	1856(26.0)	1893(6.8)	16309(58.8)	2222(6.4)	18164(52.1)	
**Time of ANC initiation**							< 0.001^a^
1^st^	446(6.2)	195(2.7)	2730(9.9)	5440(19.6)	3176(9.1)	5635(16.2)	
2^nd^	2706(37.9)	1176(16.5)	7445(26.8)	10522(37.9)	10152(29.1)	11699(33.5)	
3^rd^	1984(27.8)	633(8.9)	798(2.9)	806(2.9)	2782(8.0)	1438(4.1)	
**ANC place**							< 0.001^a^
Home	280(3.9)	25(0.4)	11120(4.0)	207(0.7)	1400(4.0)	232(0.7)	
Health Facility	4856(68.0)	1978(27.7)	9853(35.5)	16561(59.7)	14710(42.2)	18540(53.2)	
Total	5136(71.9)	2004(28.1)	10973(39.6)	16768(60.4)	18772(53.8)	16110(46.2)	

*ANC* Antenatal Care, *SBA* Skilled Birth Attendant

^a^ *p*-value generated based on Pearson chi-square statistics

### Adjusted and unadjusted predictors of home birth after suboptimal ANC uptake

Table 3 presents the crude and adjusted odds ratio and 95%CI for the association between type of place of birth and women characteristics in the suboptimal ANC group. Women in the northeast, northwest and south-south are 62%, 150% and 194% more likely to deliver at home after suboptimal ANC visit compared to their northcentral counterpart respectively (Table 3). Rural women are more than two times more likely to deliver at home after suboptimal ANC visit than urban women (cOR = 2.41, 95%CI = 2.16–2.69). After suboptimal ANC, odds of non-facility delivery are higher among Muslim compared to Christian (cOR = 3.05, 95%CI = 2.74–3.40; aOR = 1.43, 95%CI = 1.05–1.93). Women in the richer wealth quintiles are less likely to deliver at home after suboptimal ANC than those in the poorest quintiles (cOR = 0.18, 95%CI = 0.15–0.22; aOR = 0.65, 95%CI = 0.44–0.98) (Table 3). Health insurance coverage lower the odds of home births after suboptimal ANC by 78%. Women expecting 5^th^ birth are more likely to have home delivery after suboptimal ANC compared to those having first birth (cOR = 2.21, 95%CI = 1.90–2.56; aOR = 1.53, 95%CI = 1.02–2.28). Odds of home delivery is higher among women who had problem getting permission for medical care than those who don’t after receiving suboptimal ANC (cOR = 1.61, 95%CI = 1.34–1.92; aOR = 1.84, 95%CI = 1.20–2.59). Having ANC in a health facility and utilizing SBA at birth significantly reduce the odds of home delivery after suboptimal ANC by about 333 and 5 times respectively (Table 3).

**Table 3:** Adjusted and unadjusted odds ratio of the association between type of place of delivery and maternal characteristics by ANC status

Characteristics	Suboptimal ANC – Model 1		Optimal ANC – Model 2		All ANC – Model 3	
	**COR(95%CI)**	**AOR(95%CI)**	**COR(95%CI)**	**AOR(95%CI)**	**COR(95%CI)**	**AOR(95%CI)**
**Age group**						
15-24^a^	Ref	Ref	Ref	Ref	Ref	Ref
25–34	0.97(0.86–1.09)	1.21(0.89–1.63)	0.69(0.64–0.73)***	0.87(0.75–1.01)	0.69(0.66–0.74)***	0.93(0.81–1.06)
35–49	1.08(0.93–1.24)	1.26(0.84–1.88)	0.63(0.53–0.73)***	0.68(0.56–0.82)***	0.69(0.65–0.74)***	0.75(0.64–0.89)**
**Education**						
No formal education^a^	Ref	Ref	Ref	Ref	Ref	Ref
Primary	0.45(0.38–0.52)***	0.81(0.60–1.08)	0.35(0.33–0.38)***	0.85(0.73–0.98)*	0.34(0.32–0.37)***	0.84(0.73–0.96)*
Secondary	0.18(0.16–0.21)***	0.83(0.59–1.51)	0.16(0.14–0.16)***	0.73(0.62–0.86)***	0.14(0.13–0.15)***	0.74(0.64–0.85)***
Tertiary	0.07(0.05–0.10)***	0.81(0.43–1.49)	0.04(0.03–0.05)***	0.42(0.32–0.53)***	0.03(0.03–0.04)***	0.45(0.35–0.56)***
**Marital Status**						
Married^a^	Ref	Ref	Ref	Ref	Ref	Ref
Unmarried^a^	0.63(0.46–0.85)**	1.50(0.87–2.57)	0.81(0.71–0.93)**	0.90(070–1.16)	0.73(0.65–0.83)***	1.00(0.80–1.25)
**Partner education**						
No formal education^a^	Ref	Ref	Ref	Ref	Ref	Ref
Primary^a^	0.47(0.41–0.56)***	1.24(0.90–1.69)	0.34(0.31–0.37)***	0.95(0.80–1.12)	0.33(0.30–0.36)***	0.98 (0.85–1.14)
Secondary	0.25(0.22–0.29)***	0.84(0.62–1.13)	0.20(0.18–0.22)***	0.84(0.72–0.98)*	0.18(0.17–0.12)***	0.83(0.72–0.95)**
Tertiary	0.18(0.14–0.21)***	0.77(0.51–1.15)	0.13(0.11–0.14)***	0.84(0.69–1.02)	0.11(0.10–0.12)***	0.82(0.69–0.98)*
**Place of residence**						
Urban^a^	Ref	Ref	Ref	Ref	Ref	Ref
Rural	2.41(2.16–2.69)***	1.00(0.77–1.30)	3.00(2.85–3.16)***	0.92(0.81–1.02)	3.19(3.05(3.34)***	0.93(0.84–1.03)
**Region**						
Northcentral^a^	Ref	Ref	Ref	Ref	Ref	Ref
Northeast	2.94(2.54–3.39)***	1.62(1.19–2.19)**	3.28(3.02–3.57)***	1.53(1.30–1.80)***	3.41(3.18–3.66)***	1.56(1.35–1.80)***
Northwest	4.05(3.47–4.72)***	2.50(1.78–3.49)***	5.11(4.69–5.56)***	2.43(2.06–2.88)***	5.02(4.66–5.40)***	2.45(2.11–2.85)***
Southeast	0.58(0.47–0.71)***	1.51(0.97–2.35)	0.39(0.35–0.44)***	0.62(0.50–0.75)***	0.38(0.35–0.43)***	0.70(0.59–0.84)***
South-south	1.52(1.23–1.87)***	3.79(2.46–5.84)***	1.17(1.06–1.28)**	1.85(1.54–2.21)***	1.11(1.03–1.21)**	2.03(1.72–2.39)***
Southwest	0.59(0.46–0.77)***	2.94(1.86–4.64)***	0.48(0.44–0.53)***	1.18(1.01–1.38)*	0.42(0.38–0.45)***	1.27(1.09–1.47)**
**Religion**						
Christianity^a^	Ref	Ref	Ref	Ref	Ref	Ref
Islam	3.05(2.74–3.40)***	1.43(1.05–1.93)*	3.04(2.89–3.20)***	0.94(0.82–1.07)	3.34(3.19–3.49)***	0.99(0.89–1.12)
Traditional/other	3.62(2.06–6.35)***	0.66(0.25–1.70)	3.29(2.50–4.31)***	1.12(0.65–1.92)	3.68(2.91–4.67)***	0.97(0.60–1.56)
**Occupation**						
Unemployed^a^	Ref	Ref	Ref	Ref	Ref	Ref
Employed	0.79(0.71–0.89)***	0.88(0.70–1.10)	0.64(0.60–0.68)***	0.95(0.85–1.07)	0.62(0.59–0.65)***	0.93(0.84–1.03)
**Wealth**						
Poorest^a^	Ref	Ref	Ref	Ref	Ref	Ref
Poorer	0.60(0.50–0.71)***	0.92(0.68–1.24)	0.51(0.46–0.56)***	0.79(0.66–0.97)*	0.51(0.46–0.56)***	0.82(0.70–0.97)*
Average	0.35(0.29–0.41)***	0.97(0.69–1.35)	0.26(0.24–0.29)***	0.77(0.63–0.93)**	0.25(0.23–0.27)***	0.79(0.67–0.93)**
Richer	0.18(0.15–0.22)***	0.65(0.44–0.98)*	0.13(0.12–0.15)***	0.75(0.61–0.92)**	0.13(0.11–0.13)***	0.73(0.60–0.87)***
Richest	0.08(0.06–0.10)***	0.64(0.38–1.06)	0.05(0.04–0.06)***	0.60(0.47–0.75)***	0.04(0.04–0.05)***	0.59(0.48–0.73)***
**Media exposure**						
No^a^	Ref	Ref	Ref	Ref	Ref	Ref
Yes	0.46(0.41–0.51)***	0.98(0.78–1.23)	0.38(0.36–0.40)***	0.95(0.85–1.05)	0.35(0.33–0.36)***	0.94(0.86–1.04)
**Wanted pregnancy**						
Then^a^	Ref	Ref	Ref	Ref	Ref	Ref
Later	0.52(0.43–0.62)***	1.16(0.81–1.67)	0.86(0.78–0.94)**	1.17(0.99–1.39)	0.76(0.71–0.83)***	1.18(1.02–1.38)*
No more	0.53(0.39–0.71)***	1.36(0.81–2.28)	0.72(0.63–0.82)***	1.27(1.01–1.60)*	0.65(0.57–0.73)***	1.29(1.04–1.59)*
**Birth order**						
1^a^	Ref	Ref	Ref	Ref	Ref	Ref
2	1.49(1.24–1.78)***	1.38(0.97–1.97)	1.22(1.12–1.33)***	1.28(1.08–1.51)**	1.24(1.16–1.34)***	1.31(1.12–1.52)***
3	1.43(1.19–1.70)***	1.39(0.95–2.04)	1.28(1.17–1.40)***	1.29(1.07–1.54)**	1.31(1.21–1.41)***	1.32(1.12–1.55)**
4	1.56(1.29–1.87)***	1.36(0.90–2.08)	1.46(1.34–1.60)***	1.41(1.16–1.70)***	1.47(1.36–1.59)***	1.41(1.18–1.67)***
5 +	2.21(1.90–2.56)***	1.53(1.02–2.28)*	2.30(2.13–2.48)***	1.53(1.27–1.85)***	2.36(2.21–2.52)***	1.55(1.30–1.83)***
**Covered by health insurance**						
No^a^	Ref	Ref	Ref	Ref	Ref	Ref
Yes	0.22(0.13–0.36)***	1.00(0.41–2.41)	0.25(0.21–0.31)***	0.79(0.57–1.10)	0.22(0.18–0.26)***	0.81(0.60–1.11)
**Medical help-permission**						
Not a big problem^a^	Ref	Ref	Ref	Ref	Ref	Ref
Big problem	1.61(1.34–1.92)***	1.84(1.20–2.59)***	1.55(1.42–1.70)***	1.19(0.99–1.42)	1.65(1.53–1.79)***	1.32(1.12–1.55)**
**Medical help-money**						
Not a big problem^a^	Ref	Ref	Ref	Ref	Ref	Ref
Big problem	1.29(1.16–1.43)***	0.84(0.67–1.06)	1.44(1.37–1.51)***	0.95(0.85–1.06)	1.53(1.46–1.60)***	0.94(0.85–1.04)
**Medical help-distance**						
Not a big problem^a^	Ref	Ref	Ref	Ref	Ref	Ref
Big problem	1.62(1.45–1.82)***	0.99(0.78–1.27)	1.61(1.51–1.70)***	1.04(0.91–1.18)	1.78(1.69–1.87)***	1.03(0.92–1.16)
**Health decision maker**						
Partner alone^a^	Ref	Ref	Ref	Ref	Ref	Ref
Joint decision	0.53(0.47–0.59)***	0.91(0.72–1.15)	0.42(0.39–0.44)***	0.87(0.77–0.96)**	0.40(0.38–0.42)***	0.86(0.78–0.95)**
Woman alone	0.48(0.39–0.59)***	0.74(0.48–1.11)	0.44(0.40–0.48)***	1.00(0.85–1.18)	0.40(0.37–0.44)***	0.96(0.82–1.12)
**Prenatal provider**						
Unskilled^a^	Ref	Ref	Ref	Ref	Ref	Ref
Skilled	0.37(0.31–0.44)***	6.76(4.94–9.25)***	0.19(0.17–0.21)***	7.61(6.39–9.06)***	0.20(0.18–0.22)***	7.12(6.12–8.28)***
**SBA use**						
Unskilled^a^	Ref	Ref	Ref	Ref	Ref	Ref
Skilled	0.005(0.004–0.01)***	0.003(0.002–0.01)***	0.006(0.005–0.01)***	0.005(0.004–0.01)***	0.005(0.005–0.01)***	0.004(0.003–0.01)***
**Timing of ANC initiation**						
1^sta^	Ref	Ref	Ref	Ref	Ref	Ref
2^nd^	0.94(0.79–1.13)	0.69(0.49–0.97)*	1.31(1.24–1.38)***	1.04(0.94–1.16)	1.44(1.37–1.52)***	1.04(0.94–1.15)
3^rd^	1.20(1.01–1.44)*	0.65(0.45–0.93)*	1.78(1.60–1.99)***	1.16(0.93–1.43)	3.04(2.81–3.29)***	1.19((1.02–1.39)*
**ANC place**						
Home^a^	Ref	Ref	Ref	Ref	Ref	Ref
Health Facility	0.23(0.15–0.34)***	0.06(0.03–0.10)***	0.10(0.09–0.13)***	0.03(0.02–0.04)***	0.13(0.11–0.15)***	0.03(0.02–0.04)***

^a^Reference category

^***^ *p* < 0.001; **p < 0.01; *p < 0.05

### Adjusted and unadjusted predictors of home delivery after optimal ANC uptake

The adjusted and unadjusted odds and the 95%CI for the association between place of delivery and women factors are shown in Table 3. Women (35–49 years) are about 1.5 times less likely to have home birth after optimal ANC compared to the (15–24 years) (cOR = 0.63, 95%CI = 0.53–0.73; aOR = 0.68, 95%CI = 0.56–0.82). Odds of home births decrease by women level of education and wealth quintiles among those who had optimal ANC. After receiving optimal ANC, Northeast (53%), northwest (143%) and south-south (85%) women are more likely to have home birth while southeast women are 38% less likely to have home delivery compared to the northcentral women (Table 3). Media exposure almost thrice reduce odds of home birth after optimal ANC (cOR = 0.38, 95%CI = 0.36–0.40) while Odds of home birth increase by 27% among women who no longer desire pregnancy after receiving optimal ANC (aOR = 1.27, 95%CI = 1.01–1.60). Odds of home birth increase by increase in birth order among women in the optimal ANC group (Table 3). Women covered by health insurance are about 4 times less likely to have non-facility delivery after optimal ANC (cOR = 0.25, 95%CI = 0.21–0.31). Women who made health decision alone or jointly with their partner are 2.3 times less likely to have home birth compared to those whose partner made their health decision alone respectively. SBA use and health facility ANC significantly reduce odds of home birth (Table 3).

### Adjusted and unadjusted predictors of home delivery after any ANC uptake

Table 3 presents odds and 95%CI of the association between type of birthplace and women characteristics among women who had at least one ANC. Women (35–49 years) are less likely to have non-facility delivery than the (15–24 years) (cOR = 0.69, 95%CI = 0.65–0.74; cOR = 0.75, 95%CI = 0.64–0.89). Odds of home birth decrease by level of education after receiving at least one ANC. Odds of home birth is 17% and 18% less likely among women whose partner has secondary and tertiary education respectively (Table 3). Women in the northeast, northwest and south-south are 56%, 145% and 103% more likely to have home birth after having one ANC compared to those in the northcentral. Women in the South-east are less likely to have non-facility birth (cOR = 0.38, 95%CI = 0.35–0.43; aOR = 0.70, 95%CI = 0.59–0.84). Odds of home delivery decrease by women wealth class and increase by women birth order. Exposure to mass media reduce the odds of home births by 65% (cOR = 0.35, 95%CI = 0.33–0.36). Undesired pregnancy increase the odds by 18% and 29% respectively (Table 3). Odds of home birth decrease by about 5 times among women covered by health insurance after at least one ANC receipt. Those who had problem getting permission for medical care are more likely to have home delivery than those who don’t (cOR = 1.65, 95%CI = 1.53–1.79; aOR = 1.32, 95%CI = 1,12–1.55). Joint health decision making decrease the odds of home birth. Using skilled health provider at ANC and delivery significantly reduce the odds of home delivery. Those who initiate ANC in the 3^rd^ trimester are more likely to have home birth (cOR = 3.04, 95%CI = 2.81–3.29; aOR = 1.19, 95%CI = 1.02–1.39) than those that initiate in 1^st^ trimester. ANC receive at a health facility decrease the odds of home birth (Table 3).

### Barriers of facility-based delivery and differences in optimal number of ANC

Table 4 presents the Proportion of Women with/without facility-based delivery barriers and the median test of differences in optimal ANC number between the groups. 8.6% (2.7% and 5.8% in the respective suboptimal and optimal group) of women had home birth because the cost of facility delivery was too high. 2.4% had home birth because facility was not open (with difference in the group median (*p* < 0.10)) (Table 4). 16.6% (5.1% in suboptimal and 11.5% in optimal group) of the women reported distance/transport issue as the barrier to facility delivery. Lack of trust/poor service was the reason for home birth in 2.6% of the women with significant difference in median statistics (*p* < 0.001) (Table 4). 0.6% did not utilize facility at delivery due to absence of female health provider. 3.6% of women whose husband/family did not allow facility delivery had home birth. 40.5% of women (13.3% in suboptimal and 27.2% in optimal group) felt it wasn’t necessary to have facility-based delivery (with median difference at *p* < 0.001) (Table 4), while it was not customary to have facility-based delivery for about 5% of the women. 5.3% of the women stated other reason for having home birth compared to the 94.7% that did not state any other reason and the group difference in median number of ANC visit is significant at *p* < 0.001 (Table 4).

**Table 4:** Barriers of facility-based delivery among women of reproductive age (15–49 years)

Barriers	Women Percentage			Median (IQR)	Chi-square^M^ (*p*-value)
	**Suboptimal** **n(%)**	**Optimal** **n(%)**	**All ANC** **n(%)**		
**Facility cost too much**					0.59(0.443)
No	2317(27.0)	5543(64.5)	7860(91.4)	5.0 (3;7)	
Yes	233(2.7)	504(5.8)	737(8.6)	5.0 (3;7)	
**Facility not open**					3.04(0.081)
No	2493(29.0)	5902(68.6)	8395(97.6)	5.0 (3;7)	
Yes	57(0.7)	145(1.7)	202 (2.4)	5.0 (3;8)	
**Too far/no transport**					0.28(0.597)
No	2112(24.6)	5056(58.8)	7168(83.4)	5.0 (3;7)	
Yes	438(5.1)	991(11.5)	1429(16.6)	5.0 (3;7)	
**Don’t trust facility/poor service**					36.60(< 0.001)
No	2521(29.3)	5855(68.1)	8376(97.4)	5.0 (3;7)	
Yes	29(0.4)	192(2.2)	221(2.6)	7.5 (3;15)	
**No female health provider**					0.90(0.343)
No	2539(29.5)	6009(69.9)	8548(99.4)	5.0 (3;7)	
Yes	11(0.2)	37(0.4)	49(0.6)	5.0 (3;8)	
**Husband/family didn’t allow**					0.22(0.640)
No	2460(28.6)	5829(67.8)	8289(96.4)	5.0 (3;7)	
Yes	90(1.1)	218(2.5)	308(3.6)	5.0 (3;8)	
**It was not necessary**					23.68(< 0.001)
No	1406(16.4)	3713(43.1)	5119(59.5)	5.0 (3;7)	
Yes	1144(13.3)	2334(27.2)	3478(40.5)	4.0 (3;6)	
**It was not customary**					1.54(0.214)
No	2426(28.3)	5748(66.8)	8174(95.1)	5.0 (3;7)	
Yes	124(1.4)	299(3.5)	423(4.9)	5.0 (3;8)	
**Other reason**					14.17(< 0.001)
No	2449(28.5)	5689(66.2)	8138(94.7)	5.0 (3;7)	
Yes	101(1.2)	357(4.1)	458(5.3)	5.0 (4;8)	
**Total**	2550(29.7)	6047(70.3)	8597(100.0)		

^M^Chi-square generated from Median test

### Impact of facility delivery barriers on type of ANC place

**Fig. 3:**
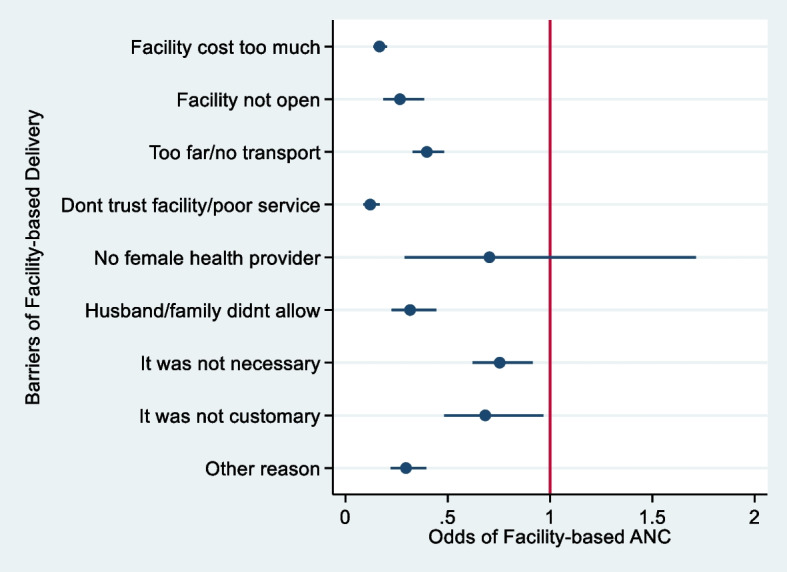
Coeff plot showing the effect of facility delivery barriers on type of ANC place

Figure 3 presents the Coeff plot for the adjusted odds of health facility use at ANC. Women who had difficulties accessing health facility at delivery are less likely to utilize health facility at ANC (aOR < 1 throughout). The odd is over 5 times less likely when difficulties such as; high facility cost (aOR = 0.16, 95%CI = 0.13–0.20) and poor facility service (aOR = 0.12 95%CI = 0.08–0.17) were reported. The odd reduces by 74% when facility was not opened (aOR = 0.26 95%CI = 0.18–0.38) (Fig. 3).

## Discussion

Women inequity and impediments factors in the use of health facility-based delivery following suboptimal and optimal utilization of pregnancy care (antenatal care) service was investigated based on analysis of data (information) collected from non-users of health facility at delivery (home birth) in a population-based survey. This is to inform on the best practice in the utilization of reproductive health service where all pregnancy matters and provide evidence-based interventional strategy to improve birth outcomes towards achieving the expected WHO standard for pregnancy care and attainment of the 2030 sustainable development goal-3 in Nigeria.

The prevalence of home birth among women who had utilized antenatal care service prior to delivery is 46.2% with 14.7% and 31.5% in suboptimal and optimal ANC visits respectively. The difference in proportion is statistically significant, and this infers that utilization of ANC in the two groups does not translate to utilization of health facility delivery and hence highlighted the major hitches in reducing maternal and child mortality in Nigeria. This agrees with findings from previous studies on; ANC barriers in Nigeria, Women attitude towards ANC service utilization in Ethiopia and whether ANC translate to skilled birth attendants utilization in Ghana [50–52].

Pregnant women utilization of non-institutional delivery service after suboptimal and optimal use of ANC service are associated with both socio-demographic and maternal health related factors that includes obstetrics and healthcare autonomous factors. The Pearson bivariate analysis evidently showcase that all the aforementioned factors are connected to maternal utilization of non-facility delivery for the suboptimal and Optimal ANC group as well as the combined group. Studies in Nigeria and other SSA have reported similar findings to be related to maternal utilization of health-facility services based on bivariate association [33, 34, 37–39, 41].

Further analysis reporting the adjusted and unadjusted association revealed that women aged 35–49 years are less likely to utilize home delivery after receiving at least one ANC service (the odds is even lower in the optimal ANC group) compared to those in the age group of 15–24 years which can be liking to the impact of maternal experience in the utilization of maternal healthcare services as women aged 15–24 years are more likely to be nursing first child [33, 53, 54]. The effect of women and partner educational level on reducing the odds of home delivery after suboptimal and optimal ANC uptake was also discovered. This agrees with recent findings in LMIC [55–58].

Geopolitical zone is associated with consistent increase and decrease in odds of home delivery after suboptimal and optimal ANC. Compared to their counterpart in the northcentral, women in the northeast, northwest and south-south are more likely to utilize home birth after ANC while those in the southeast are rather more likely to utilize health facility after ANC, as corroborated by previous studies in Nigeria [44, 52]. The effect of religion is significant in the suboptimal group as Muslim are more likely to deliver at home after suboptimal ANC compared to the Christians. Thus, pointing to the religion and socio-cultural impact on maternal healthcare service usage [59–61].

It was observed that the probability of home delivery reduces by increase in household wealth quintiles irrespective of optimality status of ANC. Hence, the richest are more likely to utilize health facility at delivery than the richer, the richer are more likely than the rich and so on. This highlights the significant of socioeconomic power in maternal healthcare delivery as reported in Nigeria and other SSA countries [8, 62, 63]. However, the chance of home delivery increase with increase in birth order whether suboptimal or optimal ANC service has been received. Thus, women with at least 5 births are more likely to utilize non-healthcare facility at delivery than those with at least 4 previous births and so on. Also, undesired pregnancy is significantly associated with home delivery after optimal ANC uptake. These were corroborated by SSA studies [60, 61, 64].

Whether women received optimal or suboptimal ANC, getting permission for medical help was consistently associated with non-utilization of health facility at delivery as those who had big problem getting permission are more likely to deliver newborn at a home. Whereas the odds of home delivery after suboptimal and optimal ANC uptake reduce significantly when women are involved in their healthcare decision with their partners (joint decision) rather than when the partner decides alone. Similar studies reported the significant of medical permission and decision-making [3, 52]. Furthermore, SBA use at delivery and ANC in a healthcare facility significantly reduce the chance of home delivery and thus appreciably raise the possibility of facility-based delivery after women had received suboptimal and optimal ANC. ANC initiated in the 3^rd^ trimester however raises the chance of home delivery after at least one ANC have been received. This agrees with recent study highlighting negative impact of delayed ANC on facility-based delivery [58].

Major barriers to utilization of facility-based delivery were cited by the non-users as reason for home delivery. 8.6% had home delivery because of high facility cost, 2.4% said facility was not opened at the time of delivery, 16.6% had no means of transport as facility was too far, poor facility service/lack of trust were reported by 2.6% of the non-users, Non-availability of female health provider was the reason for home birth among < 1% of the non-users, 3.6% stated that husband/family did not allow them to utilize health facility at delivery. As high as 40.5% (two-fifth) of the non-users felt it was not necessary to utilize health facility at delivery. About 5% added that it was not customary while reasons other than above were reported by 5.3% of the non-users. Similarly, the problem of transportation, cost and distance were told by previous studies in SSA [52, 55, 65]. There was significant difference in median number of ANC received by women between the group that felt facility delivery was necessary or not. Hence, those who felt it wasn’t necessary to deliver have had up to 4 ANC visits on the average and one fewer than those who felt otherwise. Also, women who don’t trust facility service have had more than 2 ANC visits on the average than those who trust the service. Hence the significant difference in the median number of ANC visits and thus highlights the ANC variation among non-users in Nigeria [52].

This study further determine whether the type of place of ANC can be predicted from the facility delivery barriers reported by the non-users as ANC by non-users are likely to be outside healthcare facility. Consequently, it was observed that all women who reported a form of barrier are less likely to have ANC in a healthcare facility. In fact, those who alluded to poor service and facility cost are more than 5 times less likely to utilize health facility at ANC. Non-users who reported that health facility was not opened at the time of delivery, husband/family did not allow health facility delivery and other reasons are about 3 times more likely to receive ANC service outside a healthcare facility.

### Strengths and limitations

Responder bias majorly associated with cross-sectional studies might have affected the study. However, inclusion of only Women who have had at least one ANC minimized such bias. The study findings infer associations and not causality. Hence interpretations should be limited to associations. Also, the author was limited to the choice of variables collected based on the questionnaires in the respective operationalized 2008, 2013 and 2018 NDHS. The study strengths can however be observed from the application of weighted survey data to achieve a representative sample of the target population. The fact that the study was based on 3 waves of the survey data led to a large sample size which improve the precision and reliability of the study estimates and thus the generalizability of the study findings herein. This is the first study that examine the barriers of institutional delivery based on the national survey data in Nigeria. Thus, provide evidence-based strategy for implementation of intervention to improve the coverage of health facility delivery.

## Conclusions

About half of Pregnant women had home delivery after suboptimal and optimal uptake of ANC services in Nigeria. Hence, significant disparities exist in maternal utilization of health facility at delivery with one-tenth and nine-tenth of the prevalence among the suboptimal and optimal ANC attendees respectively. Socio-demographics, obstetrics and women autonomous factors are connected to home delivery after ANC uptake. Muslim Women who had suboptimal ANC visits are more likely to deliver newborn at home compared to the Christians. Undesired pregnancy is associated with home delivery after receiving optimal ANC. Likelihood of home delivery increase with increase in parity and decrease with increase in wealth quintiles. The autonomous problem limiting women to freely seek for medical care and make health decision influence non-institutional delivery. Whereas utilization of SBA at childbirth and ANC receive in a healthcare facility predicts utilization of healthcare facility at delivery. About four-fifth of the barriers can be linked to poor facility service, cost, distance, and misconceptions. Thus, Women who alluded any form of barrier to facility delivery are more likely to have ANC outside a healthcare facility.

### Recommendations

The study findings exposed the insufficiency of ANC alone in improving overall reproductive and maternal health outcomes and thus, evidently depicts the need to strengthen all component package of the pregnancy care services. Governmental and non-governmental organization expansion of the focus antenatal care coverage is critical to improve maternal utilization of health facility at delivery. Improved patient-client service, sensitization and health education program is required to prepare women ahead of childbirth and alleviate possible barriers. Contextual research on myths and misconceptions of health facility delivery is required to understand the barriers.

## Data Availability

The de-identified data is available in the public domain and can be accessed at the open repository of the DHS program www.dhsprogram.com. The generated and/or analyzed dataset are available on reasonable request from the corresponding author.

## Abbreviations

ANC: Antenatal Care
aOR: Adjusted Odds Ratio
CI: Confidence Interval
cOR: Crude Odds Ratio
LMICs: Low- or Middle- Income Countries
NDHS: Nigerian Demographic Health Survey
NPHC: National Population and Housing Census
PRMR: Pregnancy Related Mortality Ratio
RH: Reproductive Health
SBA: Skilled Births Attendants
SDG: Sustainable Development Goal
SSA: Sub-Saharan Africa
WHO: World Health Organization
